# Neoplasms Associated with the Onset of Age-Related Macular Degeneration: A Case–Control Study Using the All of Us Cohort

**DOI:** 10.21203/rs.3.rs-8396983/v1

**Published:** 2026-02-03

**Authors:** Ethan Wu, Katherine Du, Tanner Thomas, Chris Shin, Jessica Jiang, Justin Navidzadeh, Nasiq Hasan, Joanna Yao, Michelle Zhang, José Sahel, Jay Chhablani

**Affiliations:** University of Pittsburgh School of Medicine and Medical Center; University of Pittsburgh

## Abstract

**Purpose:**

This study investigated the association between prior diagnoses of neoplasms and the onset of age-related macular degeneration (AMD).

**Methods:**

A retrospective case-control study was conducted using health records from 2 724 individuals in the All of Us Program, a longitudinal cohort of U.S. adults. Odds ratios (OR) and Fisher’s exact tests were used to evaluate associations between neoplasms and AMD in a matched cohort. Kaplan-Meier survival analysis assessed how neoplasms affected time to AMD onset.

**Results:**

Benign neoplasms of the colon (OR: 1.90, 95% CI: 1.55–2.33), skin (OR: 1.46, 95% CI: 1.16–1.84), and rectum (OR: 2.90, 95% CI: 1.53–5.48) showing the strongest and statistically significant associations with AMD. Patients with these benign neoplasms also developed AMD earlier. Although malignant neoplasms of skin of face (OR: 1.97, 95% CI: 1.11–3.49), prostate (OR: 1.47, 95% CI: 0.98–2.22), and breast (OR: 1.39, 95% CI: 0.95–2.02) were also more frequent in AMD patients, these associations were not as significant.

**Conclusion:**

Insights into the associations between neoplasms and earlier AMD onset may help ophthalmologists identify at-risk patients and enable earlier intervention. These findings suggest shared pathophysiological mechanisms, such as chronic inflammation and vascular dysfunction, may contribute to both tumour formation and AMD development.

## Summary Statement

Large scale informatic analysis applied to the All of Us Research Program uncovered novel associations between systemic benign and malignant neoplasms and age-related macular degeneration suggesting a shared multisystem basis for AMD and cancer pathogenesis.

## Introduction

Age-related macular degeneration (AMD) is one of the leading causes of blindness worldwide.^1^ It reflects the interplay between aging, environmental factors, and genetics—specifically, age, smoking, body mass index, and certain single-nucleotide polymorphisms are associated with progression to advanced AMD.^2^ The pathophysiology of AMD involves the accumulation of drusen and the degeneration of photoreceptors.^3^ While the exact mechanism of AMD is unknown, the development of AMD is associated with chronic inflammation, lipid deposition, and oxidative stress.

Interestingly, the development of cancer exhibits similar mechanistic pathways, particularly chronic inflammation and oxidative stress.^4^ Inflammatory processes can induce oxidative and nitrosative stress as well as lipid peroxidation, and the reaction of DNA with products of lipid peroxidation leads to DNA damage.^5^ Drugs that target these chronic inflammatory pathways are one of the major research avenues for effective cancer treatments.^6^

As aging is associated with reactive oxygen species formation and persistent inflammation, it may contribute to both AMD and cancer.^7–9^ Given these mechanistic similarities, emerging evidence suggests bidirectional associations between AMD and certain cancers. AMD patients show increased risk of hypervascular cancers including renal and thyroid malignancies^10^, while patients with prostate and thyroid cancers demonstrate higher AMD risk.^11,12^ Furthermore, a population-based study found that early AMD is linked to higher cancer mortality.^13^

Recently, studies have analysed large scalable datasets to show systemic disease associations and AMD.^14^ However, there does not exist a large scalable dataset study that examines the relationships between various benign and malignant types of neoplasms and AMD. The objective of this study is to provide a standardized analysis of the association between multiple different types of neoplasms and the onset of AMD. To do this, we use the *All of Us* dataset, which includes thousands of participants across the United States.

## Materials and Methods

### Study design and participants

In this retrospective study, patient data was extracted from the publicly available *All of Us* dataset (Table 1).

From the *All of Us* dataset, which includes over 256 000 patients, we identified a cohort of 1 362 patients diagnosed with wet or dry AMD using ICD-10 codes H35.32 (wet AMD) and H35.31 (dry AMD) from electronic health record (EHR) data. These patients were selected to reflect the representative distributions of ethnicity and gender in the U.S. population. Sample size was determined by the available AMD cases in the dataset.

This study adhered to the tenets of the Declaration of Helsinki. The All of Us Research Program protocol was approved by the appropriate institutional review boards, and the present analysis using de-identified data was determined by the **All of Us Institutional Review Board** to be exempt from additional review. Because only de-identified data were used, individual informed consent for this secondary analysis was not required.

To create a matched control group, 1 362 patients without any AMD diagnosis were matched 1:1 based on exact birth year, gender, and race. When multiple eligible controls existed for a single case, one was randomly selected to complete the matching.

ICD-10 codes were utilized to identify patients with AMD and associated neoplasms, as classified by the World Health Organization (WHO) in the International Statistical Classification of Diseases and Related Health Problems, 10th Revision.^15^ A comprehensive review of all ICD-10 codes related to neoplasms was performed. Only neoplastic conditions with more than 10 documented occurrences in the dataset were retained for analysis. To enhance clinical interpretability, neoplasms were subsequently categorized based on original tumour location and benign versus malignant nature.

### Comorbidity standardization

To account for natural increases in neoplasms with age and to ensure a fair comparison between cases and control, we not only age matched controls but also focused on neoplasms diagnosed before the onset of AMD. AMD onset date was defined as the earliest recorded diagnosis of either condition. For matched controls, we applied the same temporal cutoff using their matched case’s AMD diagnosis date, ensuring equal follow-up time for neoplasm development in both groups.

### Coding and analysis

Statistical analyses were performed using Python 3.12 with pandas and NumPy libraries.

### Risk Estimation in Validation Cohort

To validate the neoplasms associated with AMD, identified through supervised machine learning, we conducted a comprehensive analysis using the publicly available *All of Us* dataset, a diverse and independent cohort. The validation process involved calculating odds ratios to quantify the strength of the association between neoplasms and AMD.

Odds ratios (ORs) were calculated using the standard contingency table approach to quantify the strength of association between various benign/malignant neoplasms and AMD. For each neoplastic condition, a 2×2 table was constructed, comparing the presence or absence of the tumour in AMD patients versus healthy, non-AMD age, gender, and race-matched controls. The exact odds ratios and their corresponding 95% confidence intervals were computed using Fisher’s exact method. Additionally, *p*-values were derived to assess the statistical significance of these associations, with *p-*values less than 0.05 being statistically significant.

### Kaplan Meier Curves

To evaluate the temporal relationship between neoplastic conditions and the onset of AMD, we performed survival analyses using Kaplan-Meier curves. Time-to-event was defined as the duration from the earliest recorded encounter in the EHR to the first diagnosis of AMD. For individuals in the control group, follow-up was censored at the AMD diagnosis date of their matched case or their last encounter, whichever came first. For each validated neoplastic disease, we stratified the entire cohort into two groups based on the presence or absence of that condition and generated Kaplan-Meier curves to visualise differences in AMD-free survival. Log-rank tests were used to compare survival distributions and determine statistical significance between groups. All analyses were conducted using standard survival analysis packages in Python.

## Results

### Cohort Characteristics

This study included a total of 2 724 participants, comprising 1 362 patients diagnosed with age-related macular degeneration (AMD) and 1 362 age, race, and sex-matched controls. The cohort exhibited a balanced gender distribution, with females comprising 53.9% and males 46.1% of both groups. The racial composition was predominantly White (92.4%), with Black or African American individuals representing 6.3%, and other individuals 1.3%. The mean age across both AMD and control groups was 75.0 years (SD = 7.0 years), consistent with the expected demographic distribution of AMD incidence (Table 1).

### Association Between Neoplastic Disease and AMD

A retrospective analysis of cancer diagnoses revealed that several benign neoplasms and one malignant neoplasm were significantly associated with AMD. Notably, all neoplastic conditions examined were diagnosed prior to AMD onset, suggesting that systemic factors contributing to neoplasm development may also influence AMD pathogenesis.

**Figure 1:**
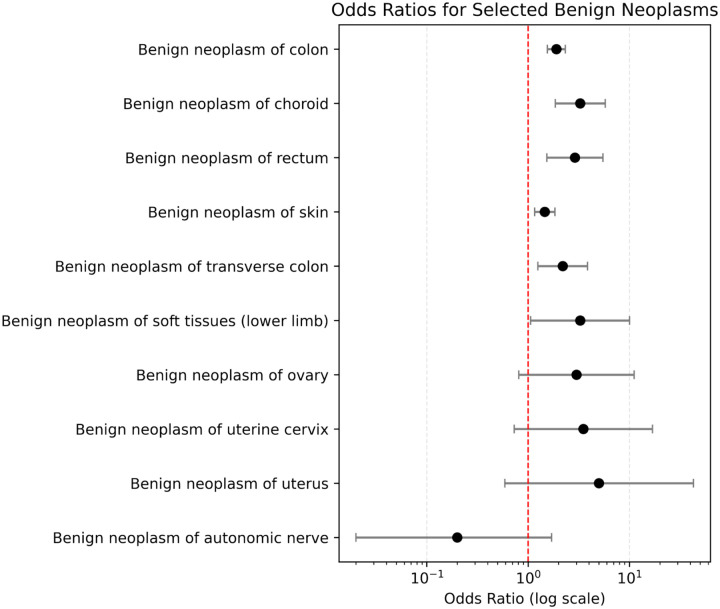
Odds Ratios of Top Benign Neoplasms and Age-Related Macular Degeneration

Benign neoplasms were significantly more prevalent in AMD patients than in controls. The strongest association was observed with benign neoplasm of the colon (OR: 1.89, 95% CI: 1.55–2.32, *p* = 4.13E-10), which occurred in 304 AMD patients compared to 179 controls. Benign neoplasms of the skin (OR: 1.46, 95% CI: 1.16–1.84, *p* = 1.27E-03) as well as benign neoplasm of rectum and anal canal (OR: 2.90, 95% CI: 1.53–5.48, *p* = 8.41E-04) also demonstrated significant associations with AMD (Table 2, Fig. 1).

**Figure 2:**
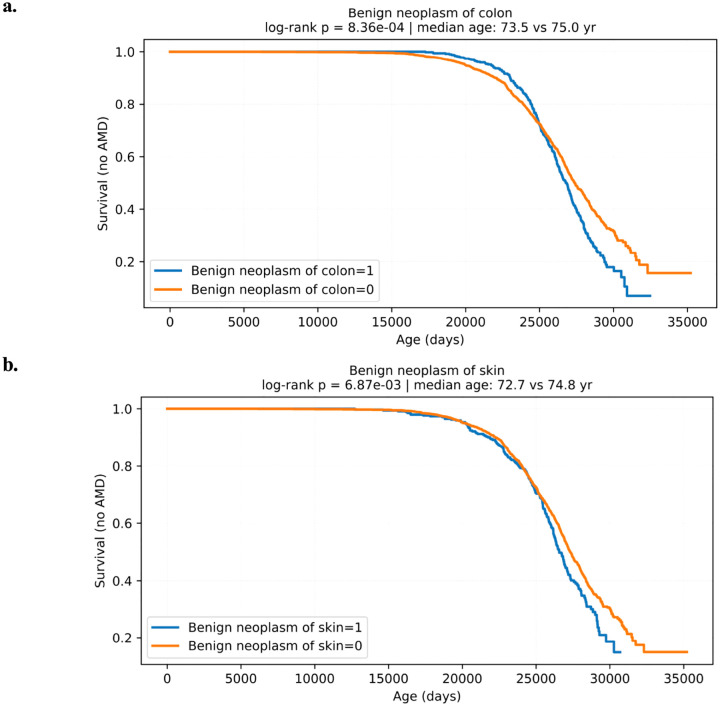
Kaplan-Meier Survival Curves of Top Neoplasms for Age-Related Macular Degeneration **Figure 2a.** Kaplan Meier Survival curve of Benign Colon neoplasm for AMD **Figure 2b.** Kaplan Meier Surivival curve of Benign Skin neoplasm for AMD

To further explore the temporal relationship between these benign neoplasms and AMD onset, Kaplan-Meier survival curves were generated. For each condition, we stratified individuals by the presence or absence of the neoplasm and tracked time from first EHR encounter to AMD diagnosis. Patients with benign neoplasms of the colon or skin showed significantly earlier AMD onset compared to those without these conditions. This separation in survival curves suggests that these comorbidities may accelerate the development of AMD or reflect underlying shared risk factors (Fig. 2).

Among malignant neoplasms, primary malignant neoplasm of the skin of the face showed a significant association with AMD (OR: 1.97, 95% CI: 1.11–3.49, *p* = 2.55E-02), occurring in 35 AMD patients compared to 1327 controls. Other malignant neoplasms showed non-significant trends toward increased odds in AMD patients, including primary malignant neoplasm of prostate (OR: 1.47, 95% CI: 0.98–2.22, *p* = 7.98E-02) and primary malignant neoplasm of female breast (OR: 1.39, 95% CI: 0.95–2.02, *p* = 1.06E-01) (Table 2)

## Discussion

In this study, we conducted a comprehensive analysis using the *All of Us* dataset, examining associations between age-related macular degeneration (AMD) and preceding diagnoses of various neoplasms. We found that benign neoplasms of the colon, skin, and rectum/anal canal were significantly more prevalent in individuals who later developed AMD, compared to matched controls, suggesting a potential link between proliferative processes and AMD pathogenesis. These associations were quantified using odds ratios and further supported by Kaplan-Meier survival analyses, which showed that patients with benign neoplasms were diagnosed with AMD at an earlier age.

The disproportionate prevalence of benign neoplasms among AMD patients suggests the potential presence of shared pathophysiological mechanisms. Chronic inflammation and vascular dysfunction are well-documented contributors to both benign tumour formation and AMD pathogenesis.^9,16^ This hypothesis is supported by evidence that benign colon neoplasms often develop within a milieu characterized by prolonged inflammatory responses and vascular remodeling.^17^ Such processes, including the release of growth factors, inflammatory cytokines, and chronic oxidative stress, are also central to AMD pathology.^18^ Therefore, one hypothesis is that systemic inflammatory and vascular changes associated with benign tumours later contribute to retinal vulnerability and degeneration.

The temporal relationship demonstrated by Kaplan-Meier survival curves further supports this hypothesis. Patients with benign colon and skin neoplasms experienced earlier AMD diagnosis compared to matched controls, indicating a possible cumulative effect of systemic pathology. This underscores the concept that AMD may not solely represent an isolated retinal pathology but rather a clinical endpoint reflecting broader systemic disturbances. Inflammation-driven tissue remodeling observed in benign neoplastic conditions could initiate or exacerbate retinal changes, facilitating earlier AMD manifestation.

The weaker association between malignant neoplasms and AMD may be explained by the more aggressive nature of malignancies, which often involve immune evasion and rapid proliferation rather than the slow, chronic remodeling seen in AMD.^19^ Additionally, many patients with a history of malignancy may undergo systemic treatments such as chemotherapy, which could alter their risk of developing AMD through immune modulation or direct vascular effects.^20,21^

This study provides compelling evidence for clinicians to recognize benign neoplasms not merely as isolated pathologies but as potential indicators of broader systemic risk factors that could impact retinal health. Early identification of these patients may enable proactive monitoring via dilated fundoscopic examination or optical coherence tomography and treatment via anti-VEGF injections or laser therapy, potentially delaying or mitigating AMD progression. Future research should focus on elucidating precise molecular pathways linking benign neoplasms and AMD, integrating genetic, proteomic, and inflammatory biomarkers to refine risk stratification and explore targeted preventive strategies.

Several limitations should be considered in this study. First, our reliance on electronic health record (EHR)-based diagnosis coding could introduce diagnostic misclassification or incomplete medical histories. Additionally, although matched for age, gender, and race, residual confounding by lifestyle or socioeconomic variables may persist. For example, the observed associations may partly reflect shared risk factors, such as diet, smoking, or metabolic syndromes, which were not fully captured in this analysis. Further prospective studies or analyses incorporating more granular clinical, genetic, and biochemical data would help validate and expand our findings.

In conclusion, our analysis provides novel insights into the relationship between benign neoplasms and AMD, highlighting systemic inflammation and vascular remodeling as key intersecting pathways. Recognizing these associations opens avenues for integrated, multidisciplinary approaches to AMD risk assessment and management, emphasizing systemic health as a crucial component of ocular disease prevention.

## Supplementary Material

Table 1 and 2 are available in the Supplementary Files section.

Supplementary Files

This is a list of supplementary files associated with this preprint. Click to download.

- table1.jpg
- Table2.jpg

## Data Availability

This study used data from the NIH All of Us Research Program. Access to the dataset is available to qualified researchers through the Researcher Workbench (https://www.researchallofus.org/) with required registration, training, and institutional approval. Analyses on age-related macular degeneration (AMD) and cancer outcomes were conducted under an approved All of Us project workspace.
